# Morphological and Anatomical Differentiation of *Potamogeton gramineus* in Relation to the Presence of Invasive Species *Elodea nuttallii*: A Case Study from Vlasina Lake, Serbia

**DOI:** 10.3390/plants13141937

**Published:** 2024-07-14

**Authors:** Danijela Nikolić, Dragana Jenačković Gocić, Irena Raca, Miodrag Đorđević, Ana Savić, Marina Jušković

**Affiliations:** 1 Department of Biology and Ecology, Faculty of Sciences and Mathematics, University of Niš, Višegradska 33, 18000 Niš, Serbia; dragana.jenackovic@pmf.edu.rs (D.J.G.); racairena@gmail.com (I.R.); anka@pmf.ni.ac.rs (A.S.); marinaju@pmf.ni.ac.rs (M.J.); 2 Department of Mathematics, Faculty of Sciences and Mathematics, University of Niš, Višegradska 33, 18000 Niš, Serbia; dmiodrag@pmf.ni.ac.rs

**Keywords:** *Potamogeton gramineus*, morphological and anatomical features, adaptations, invasion, *Elodea nuttallii*, multivariate statistics

## Abstract

*Elodea nuttallii* represents non-native and highly invasive species in Europe that significantly influence freshwater plant communities by decreasing the diversity of native species. This study aimed to determine whether the morphological and anatomical features of *Potamogeton gramineus*, a native species in Vlasina Lake, differ between sites where it coexists with *E. nuttallii* and those where *E. nuttallii* is not present. Environmental variables such as water depth, temperature, pH, conductivity, saturation, and O_2_ concentration were included in the analysis. Analyses were conducted on 32 morphological and anatomical features of *P. gramineus* collected from six sites within Vlasina Lake, comprising three sites where *E. nuttallii* was present and three sites where it was absent. The datasets containing morphometric and environmental variables underwent analysis using standard univariate techniques (Descriptive, ANOVA), Tukey’s Honest Significant Difference (HSD) test, Student’s *t*-test, and the Mann–Whitney U test, as well as multivariate statistical methods such as Canonical Discriminant Analysis (CDA). The results show the presence of morphological differentiation among *P. gramineus* individuals across the analyzed sites. These findings suggest that morphological and anatomical features, such as epidermis, mesophyll, palisade, and aerenchyma tissue thickness in floating leaves, number, length, width, and the surface area of stomata, as well as the width of submersed leaves and stem aerenchyma tissue thickness, effectively differentiate individuals that coexist with *E. nuttallii* and individuals that growth without its presence. Moreover, they indicate that *P. gramineus* exhibits a notable ability to modify its morphological traits in response to invasion.

## 1. Introduction

The impact of invasive species on native species can be diverse, often leading to predominantly negative effects [1]. This can result in reductions in population sizes and increased competitive dominance among native species, and in some cases, it may even lead to the local extinction of native species [2,3]. When an invasive species affects a native species, it often leads to alterations in four primary areas of the native’s biology: behavior, morphology, physiology, and traits associated with its life cycle [4].

The alterations in morphological traits of native species influenced by invasive species are extensively documented and confirmed across various animal species, such as native mollusk *Mya arenaria* L. [5], the Australian black snake—*Pseudechis porphyriacus* (Shaw) [6]—the lizard *Sceloporus undulates* (Bosc and Daudin) [7], and the bivalve *Anadara trapezia* Deshayes [8], as well as numerous plant species such as *Achillea millefolium* L., *Agropyron spicatum* (Pursh) A. Lӧve, *Agrostis capillaries* L., *Campanula rotundifolia* L., *Impatiens noli-tangere* L., and others [9,10]. Two particularly intriguing examples include the increase in shell breadth observed in the native bivalve *Anadara trapezia* (Deshayes) in response to altered water conditions caused by the introduced seaweed *Caulerpa taxifolia* (M. Vahl) C. Agardh [8] and the morphological changes in hind limb length noted lizards as a response to attacks by invasive fire ants [7]. In his review paper, Oduor [10] analyzed 53 studies of interactions between 20 native plant species and 10 distinct invasive plant species. The aim was to identify the ecological processes and genetic changes involved in the evolutionary responses of native species to invasion. There are two types of native species according to the history of interaction with invasive plant species: “naïve” native species, which have no known history of interactions with invasive species, and “experienced” natives [4,10,11,12]. According to Oduor [10], “experienced” native species express higher levels of growth and reproductive traits compared with “naïve”. Habitat parameters such as nutrients, light, temperature, and moisture play a crucial role in the response of native species to invasive species [13]. Daehler [14] revealed that the most prevalent growing conditions favoring native species over invaders were environments characterized by low resource availability, including nutrients, water, or light. Invasive species often compete with native species for resources such as sunlight, nutrients, and space. Native species can respond by altering their morphological characteristics to access resources more efficiently, such as adjusting root growth, leaf area, or height to enhance sunlight capture [14]. The native grass *Festuca novae-zelandiae* (Hack.) Cockayne, commonly known as hard Tusock, exhibited root growth advantages over invasive *Hieracium* spp. (hawkweeds) in specific low-fertility soils [15]. The comparative studies conducted by Marler et al. [16] and D’Antonio and Mahall [17] provided insight into the differences in root architecture between invasive and native plant species. Marler et al. [16] examined the fine-root densities of invasive *Centaurea maculosa* Lam. (spotted knapweed) and native *Agropyron spicatum* (Pursh) Scribn. and Sm. (blue bunch wheatgrass). Their findings revealed that there was no significant difference in fine-root densities when averaged across depths. However, the spotted knapweed tended to exhibit deeper roots, while blue bunch wheatgrass had denser fine roots at shallower depths. Depending on environmental conditions, either rooting strategy could be advantageous. On the other hand, the opposite rooting trend was observed in the invasive ice plant *Carpobrotus edulis* (L.) N.E.Br., with denser fine roots near the surface, whereas the native species *Haplopappus* spp. had deeper roots [17]. The comparative analysis of total leaf area and tissue construction costs between invasive and native plant species, as presented in the review paper by Daehler [14], unveils significant trends indicative of divergent ecological strategies. Invasive species tend to exhibit higher leaf area coupled with lower construction costs in comparison to their native counterparts [18].

*Elodea nuttallii* (Planch.) H. St. John (Nuttall’s waterweed) is a non-native and highly invasive submersed plant species in Europe, added to the list of invasive alien species of European Union concern [19]. *Elodea nuttallii* was first recorded in Serbia in 2006, and in Vlasina Lake specifically in 2009 [20,21,22,23]. Currently, this species dominates a substantial portion of the lake area and is actively expanding, forming a monodominant community. The negative effects of *Elodea* species on freshwater ecosystems and native species are evident in the decrease in overall macrophytes richness [24,25] and native seed banks [26], as well as alterations in plant community composition [24,27]. Experimental studies confirmed that both the spatial pattern and developmental stage of an indigenous species may influence the outcome of competition with potential invaders such as *E. nuttallii* [28]. Nutrient conditions also play an important role; when nutrient levels are high, floating species such as *Salvinia natans* (L.) All. and *Lemna gibba* L. out-compete *E. nuttallii* due to their superior ability to compete for light [29,30]. Conversely, under low nutrient concentrations, *E. nuttallii* can significantly inhibit the growth of *L. gibba* [30].

*Potamogeton gramineus* L. is a freshwater aquatic species with a native distribution across the Northern Hemisphere [31]. This species inhabits ponds, lakes, slow-moving rivers, and other aquatic habitats tolerating various water depths and nutrient levels. *Potamogeton gramineus* is an indicator of oligo-mesotrophic waters [32] and plays a significant role in aquatic ecosystems by providing habitat and food for various aquatic organisms. *Potamogeton gramineus* exhibits heterophily, a phenomenon attributed to distinct environmental conditions. The floating leaves are characterized by an ovate-lanceolate or elliptical shape, nearly round at the base or tapering into a lengthy petiole, occasionally absent. These floating leaves can reach a length of up to 7 cm and a width of up to 3 cm [33]. Submersed leaves, on the other hand, are linear-lanceolate, with tapered ends, sessile, and occasionally pointed at the apex. The length of submersed leaves ranges from 4 to 6 cm, rarely reaching up to 10 cm, with a width of up to 8 mm [33]. The margins of submersed leaves are minutely or obscurely denticulate, and the apex is mucronate, featuring 5–7 main nerves. The stems of *P. gramineus* are approximately 1.2 m in length, exhibiting branching and a rounded shape.

The present study is based on the hypothesis that the presence of the invasive species *E. nuttallii* in Vlasina Lake influences the morphological and anatomical traits of the native species *P. gramineus,* either directly or indirectly through alterations in environmental conditions. Therefore, the main aim of this investigation was to establish the nature of the morphological variability of *P. gramineus* by analyzing the morphological and anatomical features of *P. gramineus* from sites both with and without the presence of *E. nuttallii*. 

## 2. Results

### 2.1. The Morphological and Anatomical Variability of Stem, Floating, and Submersed Leaves of Potamogeton gramineus

Results of descriptive statistics for 32 morphological and anatomical features of *P*. *gramineus* individuals from six sites were presented in Table S1A,B (Supplementary Materials). 

**Figure 1:**
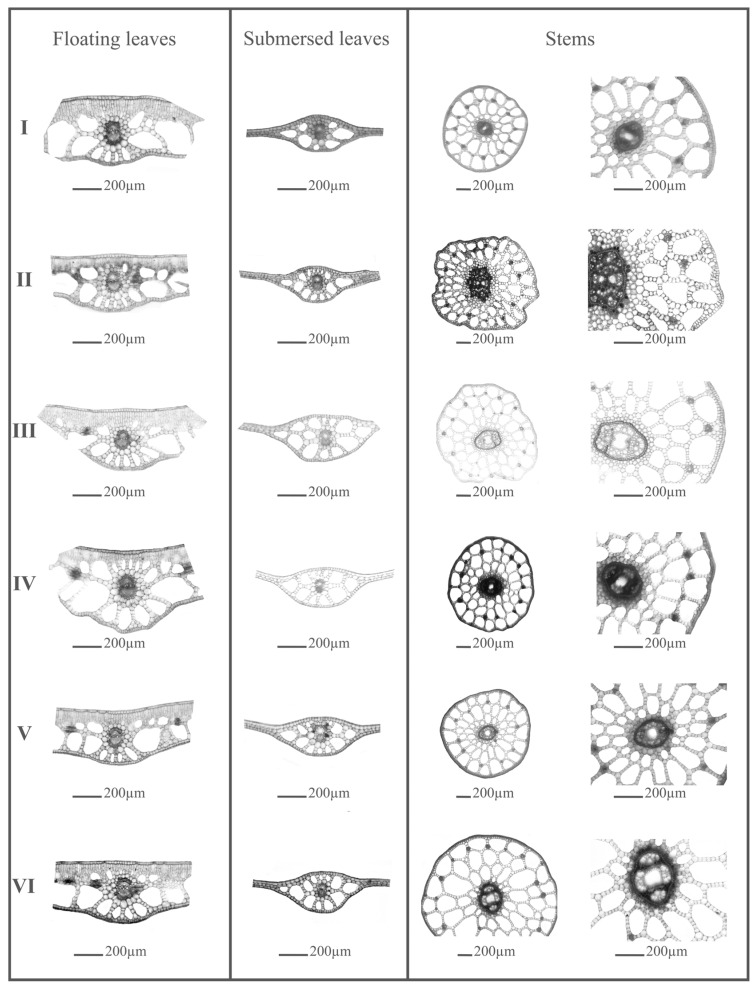
Cross-sections taken in the midrib area of the floating, submersed leaves, and stems of *P. graminues* from six sites in Vlasina Lake. In sites I, II, and III, the invasive species *E. nuttallii* was absent, while in sites IV, V, and VI, it was present.

The floating leaves of *P. gramineus* typically exhibited an ovate to elliptical shape, with either a rounded or pointed apex and a rounded base. Each leaf is attached to a petiole usually slightly longer than the blade. In a cross-section (see Figure 1), beneath the cuticle, the adaxial epidermis consisted of a single layer composed of large cells, with stomata present. Beneath the adaxial epidermis lies a multilayered mesophyll tissue composed of palisade and spongy parenchyma. The spongy tissue is characterized by large cavities forming aerenchyma, bordered by single-layer cells.

The minimal length of the floating leaf (Flo1) varied from 2.48 to 3.97 cm, while the maximum length ranged from 5.30 to 7.06 cm (Table S1A). Similarly, the minimal width of the floating leaf (Flo2) ranged from 1.06 to 1.84 cm, while the maximum ones fell between 2.23 and 3.30 cm. The largest floating leaves, according to mean values of length, width, and surface area of lamina were noticed at site I, while the smallest floating leaves were recorded at site III (Table S1A). The thickest palisade tissue (Flo7) and aerenchyma (Flo8) were observed in individuals from site I, whereas the thinnest mesophyll (Flo6) and aerenchyma were in individuals from site VI. Individuals from site III were characterized by the largest number and size of stomata (Flo10, Flo11), while those from site VI had the smallest stomata (Table S1A). The submersed leaves of *P. gramineus* typically exhibited a narrowly elliptic shape, pointed at the tip, and were usually sessile, although they may occasionally be found on a stalk. The mesophyll of the submerged leaves is underdeveloped, consisting of only a few layers with no differentiation between palisade and spongy tissues. Typically, only one leaf vein passes through the center of the leaf. Vascular bundles were encased by a single layer of cells, forming a vascular bundle sheath (refer to Figure 1).

The minimum length of a submersed leaf (Sub1) ranged from 2.67 to 4.5 cm, with a maximum length varying from 5.19 to 9.72 cm. Furthermore, the minimum width of a submersed leaf (Sub2) ranged from 0.26 to 0.61 cm, while the maximum width varied from 0.62 to 1.17 cm. The largest submersed leaves were observed at site VI, while the smallest were found at site III (Table S1B). 

The largest diameter of the stem (Stem5) was noticed at site II, whereas the smallest was found at site III. Additionally, the widest aerenchyma tissue (Ste3) was observed in individuals from site VI, while the narrowest width of aerenchyma tissue was recorded in individuals from site III. Furthermore, individuals from site I exhibited the thickest epidermis (Ste1), and both individuals from sites I and IV showed the thickest pseudohypodermis (Ste2) among the sampled populations of *P. gramineus*. 

Considering the coefficient of variability, the investigated characteristics displayed moderate-to-low degrees of variability. All morphological and anatomical features of submersed leaves and stems have shown moderate degrees of variability (10 < CV% < 50), except for the features Sub3 and Ste4, which demonstrated a very high coefficient of variability (CV > 50%) (Table S1B).

Among the morphological and anatomical features of floating leaves, three different patterns of variability were noted. The first group of characteristics (Flo1, Flo3, Flo5, Flo6, Flo7, Flo8, and Flo10) exhibited moderate coefficient of variability (10 < CV% < 50) across all sites (Table S1A). The second group consisted of characteristics with mixed variability: low (CV% < 10) in sites with *E. nuttallii* and moderate variability (10 < CV% < 50) in sites without *E. nuttallii* (Flo2 and Flo4). On the other hand, the characteristic Flo17 showed low variability in sites with *E. nuttallii* and moderate variability in sites without it. The third group included characteristics that demonstrated low variability across all six sites (Flo9, Flo15), in five sites (Flo12, Flo13, Flo14), and in four sites (Flo11, Flo16), irrespective of the presence or absence of *E. nuttallii* (Table S1A).

A one-way ANOVA was conducted to assess the impact of sample sites on morphological and anatomical features. The results reveal that all morphological and anatomical features of floating leaves, except the width of the floating leaf (Flo2), demonstrated significantly different mean values across the analyzed sites (*p* < 0.05) (Table S2, Supplementary Materials). Among submersed leaves, the statistically significant morphological and anatomical features included: their length (Sub1), width (Sub2), surface (Sub3), thickness of the mesophyll in the central part (Sub4), and diameter of the vascular bundle (Sub6). Similarly, the statistically significant morphological and anatomical features of stems were the thickness of the stem aerenchyma (Ste3) and stem diameter (Ste5). The results of the Tukey HSD post hoc test are presented in Table S2 (Supplementary Materials). The largest differences in morphological and anatomical features of floating leaves were noted between sites I and III for features Flo1, Flo3, Flo4, and Flo9 (Table S2). Individuals from site I were characterized by the largest length, surface area, thickness of the epidermis, and diameter of the vascular bundle of the floating leaves, while the individuals from site III demonstrated the smallest values of these features. Significant differences were also observed between sites I and VI for features Flo6, Flo7, Flo8, Flo9, and Flo11. As in the previous case, individuals from site I had the largest thickness of the mesophyll, palisade, aerenchyma, diameter of the vascular bundle, and surface of stomata compared with individuals from site VI. Considering the morphological and anatomical features of the submersed leaves, the largest difference was noted between sites III and VI for features Sub1, Sub 2, and Sub3. Individuals of *P. gramineus* from site I demonstrated the smallest submersed leaves, while individuals from site VI, where *E. nuttallii* was present, are characterized by the largest submersed leaves. The largest thickness of mesophyll (Sub4) was noted in site V, while the smallest thickness of mesophyll was observed in site III. Considering features Sub6, the largest differences in mean values were noted between sites III and I. The Tukey HSD post hoc test showed that the largest differences in stem features such as the thickness of aerenchyma (Ste3) were present between individuals from site III and both sites V and VI. Considering the diameter of the stem (Ste5), the individuals from site III were characterized with the smallest diameter, while individuals from sites IV, V, and VI demonstrated the largest stem diameters (Table S2).

### 2.2. The Morphological and Anatomical Differentiation within the Analyzed Samples of Potamogeton gramineus Population

According to the results of the Student’s *t*-test, statistically significant differences were noted in the following anatomical features of the floating leaves: Flo5, Flo6, Flo7, Flo8, Flo9, Flo10, Flo11, Flo12, Flo13, Flo14, Flo15, Flo16, and Flo17 (Table S3, Supplementary Materials). Higher values for these features were observed at sites without *E. nuttallii* compared with sites with *E. nuttallii*. Regarding morphological and anatomical features of submersed leaves and stems, only features Sub2 and Ste3 showed statistically significant differences, with higher values noted at sites with *E. nuttallii* (Table S3).

**Figure 2:**
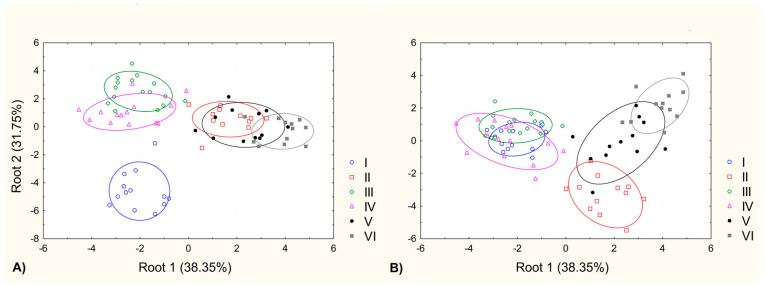
Results of Canonical Discriminant Analysis (CDA) based on all morphological and anatomical features of *Potamogeton gramineus* population: (**A**) scatterplots with canonical scores for each individual in a space defined by the first and second CDA axes; (**B**) scatterplots with canonical scores for each individual in a space defined by the first and third CDA axes.

Canonical Discriminant Analyses (CDA) resulted in two distinct morphological groups, delineated by the first CDA axis (Root1), which explained 38.35% of the total differentiation (Figure 2A). Individuals from sites IV, V, and VI clustered in the positive part of the first CDA axis, while those from sites I, II, and III were positioned in the negative part. Site I notably stood out in the negative part, clearly differentiated from all other sites, along the second CDA axis (Root2), which explained 31.75% of the total differentiation (Figure 2A). When considering the scatter plot defined by the first and third CDA (Root3) axes (15.94% of total differentiation), two discernible groups emerged: one comprising individuals from sites IV, V, and VI and the other including individuals from sites I, II, and III. Along the third CDA axis, differentiation was observed primarily between sites IV and VI (Figure 2B).

The results of the Mann–Whitney U test reveal that temperature and pH values were the most significant environmental variables responsible for differentiating sites where *E. nuttallii* was present from those where it was absent (Table S4, Supplementary Materials). Higher temperature values were observed at sites without *E. nuttallii*, whereas higher pH values were found at sites where *E. nuttallii* was present (Table S4).

## 3. Discussion

Aquatic plant habitats face numerous threats, including eutrophication, rising water temperatures, and the introduction of exotic invasive species [34]. One response of aquatic plants to these variable conditions is extensive morphological variability observed in many species from the genus *Potamogeton* [35,36,37]. On the other hand, there are some species from the genus *Potamogeton* (*P. perfoliatus* L. and *P. nodosus* Poir.) that cannot tolerate a drought period longer than 5 days [38].

*Potamogeton gramineus* has been the subject of several investigations regarding its morphological response to different environmental variables. Spencer and Ksander [35] investigated the influence of planting depth on morphology and growth, concluding that *P. gramineus* thrived when planted in shallow depths; however, when winter buds were planted at a depth of 20 cm, they showed a lower growth rate. Additionally, Spencer and Ksander [36] examined the effects of temperature and light on the early growth of *P. gramineus*, revealing that temperature and light strongly influenced elongation rate and biomass production, although their influence was not linear. Specifically, at very low light levels, increasing temperature led to decreased dry weight, while at higher light levels, increasing temperature resulted in higher rates of dry weight increase [36]. In contrast, Bayindir and Ikinci [39], in their study on the role of environmental factors in the distribution of *Potamogeton* species, reported that *P. gramineus* was positively correlated with pH and water temperature and negatively correlated with electroconductivity. Furthermore, Sondergaard et al. [40], in their research on submerged macrophytes as indicators of the ecological quality of lakes, noted that *P. gramineus* is an indicator of nutrient-poor conditions.

The anatomical features of floating and submersed leaves and stems have not been previously investigated in the referenced studies, rendering direct comparisons with our findings impractical. However, we can juxtapose our measurements of floating and submersed leaves of *P. gramineus* with data reported in established botanical references such as the Flora of Serbia [33], Flora Europaea [41], and Floras of neighboring countries like Bulgaria [42], Romania [43], and Hungary [44]. These sources provide valuable insights into the typical dimensions of *P. gramineus* leaves. The most frequently reported dimensions for floating leaves were 7cm in length and 3cm in width [33,41,42,43]. A study by Soó [44] presented a range for floating leaf length (2.5–7cm) and width (0.8–2cm) aligning closely with our results. Conversely, our study revealed slight discrepancies in the dimensions of submersed leaves compared with previous research. While Janković and Topa [33,43] reported lengths ranging from 4 to 6 cm (10) and widths up to 8 mm, our measurements indicate lengths varying from 2.67 to 9.71 cm and widths from 0.26 to 1.17 cm. Furthermore, publications by Yordanov [42], Topa [43], and Soó [44] delineated several varieties of *P. gramineus* including *P. gramineus* var. *lacustris* Fr., *P. gramineus* var. *fluviatilis* Fr., *P. gramineus* var. *heterofilus* (Schreb.) Fr., *P. gramineus* var. *stagnalis* Fr., *P. gramineus* var. *platyphylus* Rchb., *P. gramineus* var. *amphibious* Fr., *P. gramineus* var. *riparius* Fr., and *P. gramineus* var. *terestris* Schltdl. These varietal descriptions underscore the considerable morphological variability inherent in this species.

Our study revealed significant variability in the morphological and anatomical features of leaves and stems across sampling sites. Nearly all morphological and anatomical features of floating leaves significantly distinguished individuals of *P. gramineus* at sites where *E. nuttallii* was present from those collected at sites where *E. nuttallii* was absent. The individuals from sites without *E. nuttallii* were characterized by the smallest floating leaves, thickest mesophyll, palisade, aerenchyma, and epidermal layers, accompanied by the highest number of largest stomata (Table S3). Statistically significant differences in temperature values were noted among the sampled sites with and without *E. nuttallii*, with higher values recorded at sites without *E. nuttallii* (Table S4). These environmental variables likely contributed to the reduction in floating leaf area, potentially optimizing photosynthetic efficiency in response to elevated temperatures while simultaneously decreasing transpiration rates [45]. It is well known that photosynthesis is a chemical reaction catalyzed by enzymes, and its rate is dependent on leaf temperature [46]. The rate of photosynthesis increases with rising temperatures up to an optimum point [47]. Beyond this optimal temperature, the photosynthetic rate declines. Leaf size plays a crucial role in regulating temperature. An increase in leaf area thickens the boundary layer, a stagnant air layer surrounding the leaf [48]. This thickening increases resistance to water and CO_2_ diffusion [48], resulting in slower evaporation rates and reduced CO_2_ exchange between the leaf and the atmosphere [45,49]. Okajima et al. [45] investigated the influence of leaf size, temperature, and wind speed on photosynthetic rate and confirmed that at warmer temperatures, the increase in leaf temperature does not lead to an increase in photosynthetic rates. Therefore, a smaller leaf size seems more advantageous for efficient photosynthesis. Bayindir and Ikinci [39] found that *P. gramineus* has high temperature tolerance (4.95 °C), with an optimum temperature of 26.5 °C, and low tolerance for pH (0.15), preferring a higher pH optimum of 8.24. Additionally, *P. gramineus* occurred in waters with the lowest EC optima (271 μS/cm^−1^) [39].

Szabo et al., [29] confirmed that the presence of *Elodea* increases pH values in water and under lower nutrient conditions, strongly reduces the growth of the floating plant *L. gibba.* Our study demonstrated significant differences in pH values between sites with and without *E. nuttallii*, with higher pH values noted at sites where *E. nuttallii* was present (Table S4). *Elodea nuttallii* is very resilient to the immense shading caused by floating plants [50], which is advantageous in the case of the presence of *P. gramineus* floating leaves above *E. nuttallii* individuals. At sites where *E. nuttallii* was present, individuals of *P. gramineus* were characterized by the broadest submersed leaves, and thickest stem aerenchyma (Table S3). These characteristics indicate the tolerance strategy of *P. gramineus* in the presence of the invasive species *E. nuttallii*, primarily by maximizing leaf size and aerenchyma thickness, as also reported by Puijalon et al. [51]. The increased size of the submersed leaves in *P. gramineus*, caused by the dense stands of *E. nuttallii*, is a response to decreased light availability. The reduction in the total number of leaves while maximizing surface area for light capture is also observed in shade-inhabiting species such as *Polygonum caespitosum* Blume [52]. On the other hand, dense macrophyte growth *of E. nuttallii* can decrease oxygen levels due to the accumulation of organic detritus and fine sediment, as stated by Kaenel et al. [53]. The lower oxygen concentration in the presence of *E. nuttallii* might contribute to the thickened aerenchyma tissue in the submersed leaves of *P. gramineus* individuals. This adaptation is similar to what is observed in species like *Pontederia azurea* (Sw.) Kunth and *P. crassipes* Mart., which grow in temporary ponds with low dissolved oxygen concentration [54]. It is evident that morphological and anatomical differentiations of *P. gramineus* exist in Vlasina Lake (Figure 2A,B), but the reasons for these variations are still unknown.

Individuals of *P. gramineus* in sites where *E. nuttallii* was present are characterized by thinner floating leaves with the lowest surface area of stomata and by the broadest submersed leaves. Stem aerenchyma is larger in individuals of *P. gramineus* from sites where *E. nuttallii* was present compared with individuals from sites where it was absent.

This suggests that the morphological and anatomical characteristics used in this study are effective in distinguishing between the individuals of *P. gramineus* from different sites and that the presence or absence of *E. nuttallii* may reflect these changes. However, the influence of environmental variables such as light and nutrients on the morphology of this species cannot be ignored, and future experimental studies in controlled conditions are needed.

## 4. Material and Methods

### 4.1. Study Area

Vlasina Lake is an artificial water body located within a protected area known as the Natural Asset of Exceptional Importance and a Ramsar site. It ranks as the second-largest man-made lake in the southeastern region of the Republic of Serbia [55]. At an altitude of 1213 m a.s.l., this lake represents the highest-altitude lake in the Republic of Serbia. It stretches 9 km in length, with an average width of 1.8 km (maximum 3.5 km), a depth of up to 35 m, and an indented shore spanning 132.5 km. The surface area of Vlasina Lake covers approximately 16 km^2^ [56]. According to a recent investigation of water quality using the Serbian Water Quality Index, based on parameters including oxygen saturation (OS), biochemical oxygen demand (BOD), ammonium (NH_4_-N), pH, total nitrogen oxides (TNO), orthophosphate (PO_4_^3−^), suspended solids (SS), temperature (T), conductivity, and coliform bacteria (CB), this lake is characterized by good-to-excellent water quality. It represents clean water suitable for tourism and recreation [55]. Simić et al. [57] classified Vlasina Lake as an oligotrophic lake according to Carlson’s Trophic State Index (TSI) based on chlorophyll a (Chl a) concentrations.

### 4.2. Environmental Variables

The temperature, pH, conductivity, dissolved oxygen, and oxygen saturation were measured in the field using a WTW^®^ Multi 340i probe, while depth was measured using an Arhus tape measure. The water depth varied across sites, but all physical and chemical parameters were measured at the same depth using sensors or electrodes of the WTW multi 340i multimeter, positioned close to the surface of the water. Detailed information about sampling localities and measured physical and chemical parameters are presented in Table 1.

### 4.3. Plant Material

On 18 September 2020, six samples comprising a total of 79 individuals from 6 subpopulations of *P. gramineus* were collected from Vlasina Lake (Figures S1 and S2, Supplementary Materials). The presence of the invasive species, *E. nuttallii*, was taken into consideration during the collection of plant material. In sites I, II, and III, the invasive species *E. nuttallii* was absent, while in sites IV, V, and VI, it was present in varying amounts quantified as dry biomass per m^2^ (Table 1). The plant material was preserved in 50% ethanol for morphological and anatomical study. All sites were located on the same side of the lake, with distances of approximately 100 m between them.

To determine the dry biomass of *E. nuttallii* and *P. gramineus*, individuals of both species were sampled in the littoral area of the lake near the shore along six transects. Sampling was conducted using 0.25 m^2^ quadrats spaced at 5 m intervals. The plant material was placed into a nylon bag and then transferred to the laboratory. Three subsamples of *P. gramineus* and *E. nuttallii* taken from one transect were combined into one representative sample. The plant material was dried at 60 °C for 24 h in an oven until it reached a constant mass and then weighed to estimate the dry biomass [58].

The identification of the plant material was performed according to the Flora of Serbia, volume VII [33], and Flora Europaea, volume 5 [41]. The plant material is deposited under its original voucher numbers (HMN18344; HMN18622) in the Herbarium Moesiacum Niš (HMN), University of Niš, Faculty of Sciences and Mathematics, Serbia.

### 4.4. Morphological and Anatomical Analysis

Morphological and anatomical analysis was conducted on floating and submerged leaves, as well as stems belonging to 79 different individuals (32 quantitative morphological and anatomical features) (Table 2).

Morphological features of floating and submersed leaves referring to shape and size (length, width, and surface) were measured (Table 2). For this purpose, leaves were placed and pressed on paper, photographed, and then measured using ImageJ software version 1.46 (Figure S3, Supplementary Materials) [59]. For the investigation of the anatomical features of leaves and stems, cross-sections of the middle region of leaves and stems were made using manual microtome [60]. Epidermal structure and stomata were investigated on the leaf cuts, which were treated with a solution consisting of hydrogen peroxide and potassium hydroxide for three to six days to elucidate the tissue [61,62]. Temporary slides were photographed using the Leica DFC490 camera configured on the Leica DM 1000 fluorescence microscope (DM1000-FL-HI, Leica Microsystems©, Wetzlar, Germany) and examined. The anatomical features were measured using ImageJ software, version 1.46 [59]. The list of measured morphological and anatomical features is presented in Table 2.

### 4.5. Statistical Analyses

Descriptive statistics, including minimum, maximum, mean, standard deviation, and coefficient of variability were calculated for all 32 quantitative morphological and anatomical features. The coefficient of variability was used to describe the morphological variability of features. Highly morphologically variable features had CV% > 50, and moderately variable features fell within the range of 10 < CV% < 50, while stable features had a coefficient of variation below 10%. After confirming the normality of the data sets for measured features, one-way Analysis of Variance (ANOVA) was used to test the significance of differences in morphological and anatomical traits between different sites. Tukey’s Honest Significant Difference (HSD) test was performed to assess the significance of differences between pairs of group means.

To compare the morphological and anatomical features of individuals from sites where *E. nuttallii* was present and those where it was absent, we used an independent sample Student’s *t*-test.

Canonical Discriminant Analysis (CDA) was performed on the 32 morphological and anatomical quantitative features to determine the morphological and anatomical differentiation among sites. Canonical scores for each case were calculated with the aim of measuring the distances between individuals, and a scatter plot of canonical scores was generated to visualize the relationships between samples.

To assess significant statistical differences between sites with the presence of *E. nuttallii* and those without it in terms of environmental variables, the Mann–Whitney U test was performed due to the non-normal distribution of data (with only 3 data points per group).

The descriptive statistics, ANOVA, Tukey’s Honest Significant Difference (HSD) test, and CDA statistical analyses were computed using STATISTICA 7.0 software [63]. Student’s *t*-test and the Mann–Whitney U test were conducted using SPSS software (IBM, Version 19) [64].

## 5. Conclusions

Correlating the morphological and anatomical features of *P. gramineus* with the presence of the invasive species *E. nuttallii* provides valuable insights into the adaptive strategies of *P. gramineus* and its interactions with its surroundings. For a better understanding of the nature of morphological and anatomical variability in *P. gramineus*, the future investigations should include a larger number of sites and incorporate more environmental variables. The ability of *P. gramineus* to successfully coexist with *E. nuttallii* suggests the possibility of using this species in the restoration of invaded freshwater ecosystems.

## Figures and Tables

**Table 1:** List of sites with physical and chemical parameters of water where samples from the population of *Potamogeton gramineus* were collected, along with the dry biomass of analyzed species.

Sites	Coordinates	Depth (cm)	Теmperature (°C)	pH	Oxygen Saturation (%)	Conductivity (µS/cm)	Oxygen Concentration (O_2_ mg/L)	Dry Biomass *P. gramineus/E. nuttallii* (g/m^2^)	Number of Individuals
I	42°44′37″ N 22°20′13″ E	56	22.2	6.1	121.5	84.8	8.8	242.66/0	13
II	42°44′09″ N 22°20′27″ E	64	23.5	5.9	145.8	82.5	11.6	132.13/0	12
III	42°44′15″ N 22°20′21″ E	34	26	5.98	132	83.3	8.5	211.6/0	16
IV ^*^	42°44′31″ N 22°20′07″ E	48	21.8	6.51	137.2	84.8	11	126.4/20	14
V ^*^	42°44′12″ N 22°20′45″ E	78	20.8	6.5	108.8	85.5	8.42	187.33/35.3	12
VI ^*^	42°44′02″ N 22°20′38″ E	57	20.6	6.6	117.4	85.8	8.98	67.2/133.06	12

* The list of sites where the *Elodea nuttallii* was present.

**Table 2:** List of measured morphological and anatomical features of *Potamogeton gramineus* along with their corresponding codes and units.

Code	Morphological and Anatomical Features	Units
Flo1	Length of the floating leaf lamina	cm
Flo2	Width of the floating leaf lamina	cm
Flo3	Surface area of the floating leaf lamina	cm^2^
Flo4	Thickness of the adaxial epidermis of the floating leaf	µm
Flo5	Thickness of the abaxial epidermis of the floating leaf	µm
Flo6	Thickness of the mesophyll tissue of the floating leaf	µm
Flo7	Thickness of the palisade tissue of the floating leaf	µm
Flo8	Thickness of the aerenchyma tissue of the floating leaf	µm
Flo9	Diameter of the vascular bundle of the floating leaf	µm
Flo10	Number of stomata on the adaxial side of the floating leaf	/
Flo11	Surface area of stomata on the adaxial side of floating leaf	µm^2^
Flo12	Length of stomata of the adaxial side of the floating leaf	µm
Flo13	Width of stomata of the adaxial side of the floating leaf	µm
Flo14	Surface area of the epidermal cells on the adaxial side of the floating leaf	µm^2^
Flo15	Surface area of the epidermal cells on the abaxial side of the floating leaf	µm^2^
Flo16	Length of the epidermal cells on the adaxial side of the floating leaf	µm
Flo17	Length of the epidermal cells on the abaxial side of the floating leaf	µm
Sub1	Length of the submerged leaf lamina	cm
Sub2	Width of the submerged leaf lamina	cm
Sub3	Surface area of the submerged leaf lamina	cm^2^
Sub4	Thickness of the mesophyll in the central part of the submerged leaf	µm
Sub5	Thickness of the mesophyll in the lateral part of the submerged leaf	µm
Sub6	Diameter of the vascular bundle of the submerged leaf	µm
Sub7	Surface area of the epidermal cells of the submersed leaf	µm^2^
Sub8	Length of the epidermal cells of the submersed leaf	µm
Sub9	Width of the epidermal cells of the submersed leaf	µm
Ste1	Thickness of the stem epidermis	µm
Ste2	Thickness of the stem pseudo-hypodermis	µm
Ste3	Thickness of the stem aerenchyma	µm
Ste4	Thickness of the stem endodermis	µm
Ste5	Diameter of the stem	µm
Ste6	Diameter of the stem stele	µm

## Data Availability

Data are contained within the article and Supplementary Materials.

## Supplementary Materials

The following supporting information can be downloaded at: https://www.mdpi.com/article/10.3390/plants13141937/s1, Figure S1. *Potamogeton gramienus* stands in Vlasina Lake. Figure S2. *Elodea nuttallii* stands in Vlasina Lake. Figure S3. The floating and submersed leaves of *Potamogeton gramineus*. Table S1A. Results of descriptive analysis for the dataset comprising morphological and anatomical features of the floating leaves of *Potamogeton gramineus.* Table S1B. Results of descriptive analysis for the dataset comprising morphological and anatomical features of the submersed leaves and stems of *Potamogeton gramineus.* Table S2. The results of ANOVA test and Tukey HSD test for the dataset comprising morphological and anatomical features of the floating, submersed leaves, and stems. Table S3. The results of independent samples Studens´s t-test for the dataset comprising all measured morphological and anatomical features of *Potamogeton gramineus*, Table S4. The results of Mann Whitney U test comprising the environmental variables measured at sites with and without present *Elodea nuttaliii*.
