# Supplementary material for: Morphological and Anatomical Differentiation of Potamogeton gramineus in Relation to the Presence of Invasive Species Elodea nuttallii: A Case Study from Vlasina Lake, Serbia
Source: Plants (Basel). 2024 Jul 14;13(14):1937. doi: 10.3390/plants13141937 (PMC11280814; doi:10.3390/plants13141937)
Supplement: Supplementary file 1 [file plants-13-01937-s001.zip › Table S1A na S1B.pdf]

Table 3A. Results of descriptive analysis for the dataset comprising morphological and anatomical features of the floating leaves of *Potamogeton gramineus.*

Site		Flo1	Flo2	Flo3	Flo4	Flo5	Flo6	Flo7	Flo8	Flo9	Flo10	Flo11	Flo12	Flo13	Flo14	Flo15	Flo16	Flo17
I	Mean	5.51	2.10	8.79	13.33	12.99	336.87	141.91	204.36	181.95	20.69	515.64	32.29	15.98	331.07	277.68	30.75	27.08
	Min	2.50	1.06	2.00	10.78	9.96	215.06	96.48	113.53	139.97	14.00	480.56	30.85	14.55	299.19	244.45	27.15	23.25
	Max	7.06	3.30	16.17	18.58	20.42	466.27	196.74	264.73	195.80	25.00	601.38	34.21	17.81	371.84	310.25	33.87	29.14
	CV(%)	22.27	30.95	45.31	18.67	23.70	19.34	21.60	22.24	8.61	16.67	5.70	3.74	5.31	7.25	8.63	7.85	6.78
II	Mean	4.80	1.94	6.82	13.16	12.98	250.88	118.90	133.48	168.92	19.50	570.38	33.76	20.11	319.28	287.55	28.01	25.46
	Min	3.78	1.23	3.54	7.77	8.19	188.10	78.64	80.65	149.70	14.00	445.59	29.98	17.65	261.84	244.12	23.71	21.55
	Max	6.36	2.66	12.25	18.44	18.00	438.63	174.26	274.20	203.34	23.00	677.75	35.65	23.71	364.83	322.16	31.86	29.58
	CV(%)	15.10	22.04	35.83	24.04	27.42	24.09	25.48	38.56	9.34	14.33	13.22	4.97	9.63	8.90	9.62	8.53	10.34
III	Mean	4.30	1.80	5.43	10.58	11.02	260.37	115.62	148.27	148.06	21.31	588.90	34.00	20.47	318.85	291.63	28.17	25.16
	Min	2.48	1.12	2.46	6.64	7.97	226.03	71.09	103.25	130.27	13.00	450.33	29.69	17.36	261.87	244.17	23.69	21.15
	Max	5.32	2.23	8.15	16.18	18.61	293.20	171.38	184.43	169.31	28.00	650.28	35.96	23.77	364.14	322.52	31.78	29.89
	CV(%)	19.50	16.82	32.30	24.43	21.89	7.10	23.62	16.35	7.09	17.45	8.76	5.09	10.06	8.29	9.66	7.95	11.02
IV	Mean	4.55	2.00	6.67	10.91	9.90	250.48	109.18	140.06	165.24	17.00	481.70	30.15	15.57	317.16	260.82	26.51	23.26
	Min	3.77	1.71	4.35	7.96	7.74	192.78	88.07	103.79	146.31	10.00	442.22	27.99	14.36	299.47	230.21	24.45	21.18
	Max	5.78	2.33	9.27	13.26	12.38	307.31	142.89	192.01	180.28	25.00	540.21	32.01	18.21	335.11	300.13	29.44	25.69
	CV(%)	16.01	9.92	24.70	14.69	11.81	13.92	17.87	19.78	6.58	31.23	7.02	4.33	7.92	3.75	10.15	5.99	5.96
V	Mean	4.59	1.99	6.54	12.12	10.99	251.47	111.06	143.38	158.26	16.75	489.49	27.81	17.86	291.16	260.92	26.67	23.30
	Min	3.82	1.68	4.22	10.14	8.47	205.84	83.76	94.23	129.89	10.00	415.36	21.25	15.92	250.69	230.32	24.63	21.08
	Max	5.30	2.53	9.23	13.93	12.54	310.77	155.08	187.16	181.41	23.00	601.17	33.65	21.85	331.51	300.67	29.03	25.89
	CV(%)	11.53	13.04	24.58	10.06	13.04	14.63	21.27	19.49	10.57	26.84	10.44	15.04	9.21	10.59	10.16	5.37	5.81
VI	Mean	5.03	2.19	7.76	12.64	11.39	208.97	80.49	130.52	144.00	19.08	478.36	27.42	17.47	317.38	295.21	29.43	26.36
	Min	3.97	1.84	5.40	11.32	9.43	143.41	60.85	83.02	128.65	12.00	420.65	25.01	15.78	299.56	255.97	27.78	23.96
	Max	6.24	2.49	10.20	13.34	13.34	273.59	113.21	183.02	175.97	24.00	520.02	31.02	19.81	335.21	310.13	32.22	29.54
	CV(%)	14.44	9.96	21.24	7.27	12.00	19.04	20.88	26.23	9.19	18.62	6.63	6.94	5.70	3.79	5.89	4.99	6.85

Table 3B. Results of descriptive analysis for the dataset comprising morphological and anatomical features of the submersed leaves and stems of *Potamogeton gramineus.*

Site		Sub1	Sub2	Sub3	Sub4	Sub5	Sub6	Sub7	Sub8	Sub9	Ste1	Ste2	Ste3	Ste4	Ste5	Ste6
I	Mean	5.60	0.59	2.74	254.54	47.67	102.16	265.36	20.48	11.10	6.72	14.49	530.45	9.03	1310.73	199.41
	Min	2.67	0.26	0.60	169.81	40.02	80.07	155.28	11.46	5.86	4.05	6.68	388.52	4.05	955.11	147.25
	Max	8.65	1.17	6.77	282.29	56.64	117.53	351.55	29.01	15.03	13.45	19.65	682.49	14.43	1626.18	261.52
	CV (%)	33.31	44.21	60.98	12.58	9.95	10.20	22.26	27.29	27.82	39.03	28.98	17.17	32.61	13.79	17.14
II	Mean	5.22	0.70	2.53	254.52	51.18	98.79	268.76	19.44	11.36	5.81	13.36	535.53	8.01	1428.29	231.57
	Min	3.75	0.54	1.04	208.12	43.86	82.10	155.97	12.08	7.86	3.38	10.72	377.14	5.01	1176.39	168.69
	Max	6.65	0.89	3.92	307.77	61.35	110.89	380.71	26.18	15.98	8.14	18.23	728.01	13.12	1732.21	353.95
	CV (%)	17.34	16.11	30.20	10.90	12.17	9.02	21.34	28.90	23.71	28.52	18.10	15.95	30.23	12.36	20.86
III	Mean	4.27	0.52	1.60	196.37	44.85	79.84	272.24	21.05	11.29	5.13	13.16	425.81	10.28	1112.49	185.70
	Min	3.00	0.40	0.90	153.27	33.59	64.62	115.92	11.08	7.18	2.03	8.35	365.03	4.77	954.36	130.94
	Max	5.19	0.62	2.19	306.26	55.47	105.84	380.29	29.89	17.45	10.13	18.34	568.85	16.33	1542.74	325.98
	CV (%)	12.46	12.95	20.77	19.18	12.79	13.07	27.23	30.83	24.42	39.02	24.21	13.65	24.45	15.40	26.59

IV	Mean	4.49	0.57	1.82	218.56	54.19	86.69	280.88	20.70	9.27	5.15	12.43	526.32	10.73	1371.21	233.46
	Min	2.88	0.45	0.97	176.97	43.18	74.54	228.52	11.15	6.96	2.58	9.11	346.71	4.88	1027.66	170.28
	Max	5.75	0.73	2.87	278.22	106.33	98.68	370.71	31.01	12.35	7.32	17.99	712.58	17.07	1690.46	313.31
	CV (%)	19.64	15.49	35.45	18.39	31.84	8.49	16.20	31.20	19.75	26.99	18.86	20.92	33.74	16.07	18.20
V	Mean	4.54	0.71	2.53	264.98	51.23	97.42	265.95	19.44	10.87	5.39	13.03	570.79	9.57	1407.89	188.47
	Min	3.07	0.50	1.18	174.56	38.83	76.94	155.92	13.08	5.73	2.99	7.47	379.67	7.15	1089.85	118.51
	Max	5.68	0.82	4.34	295.36	64.03	126.06	350.55	29.01	15.95	8.14	19.19	778.08	12.64	1831.83	250.02
	CV (%)	21.41	16.29	34.26	13.50	15.83	13.00	24.28	27.54	26.18	29.16	23.87	23.04	15.04	18.27	22.43
VI	Mean	6.05	0.80	3.52	235.44	48.80	97.26	280.12	20.99	9.75	5.11	14.58	595.40	8.93	1386.26	210.81
	Min	4.50	0.61	2.04	150.25	42.19	70.72	201.12	12.08	6.39	2.40	10.57	413.23	3.60	1098.72	138.25
	Max	9.71	0.97	6.13	291.09	59.22	132.09	369.71	29.13	13.98	10.06	24.51	1129.50	21.68	2120.54	487.74
	CV (%)	24.54	13.43	31.72	21.63	12.52	18.73	17.55	26.58	22.60	40.67	30.77	38.61	53.08	26.86	46.32
