# Supplementary material for: Morphological and Anatomical Differentiation of Potamogeton gramineus in Relation to the Presence of Invasive Species Elodea nuttallii: A Case Study from Vlasina Lake, Serbia
Source: Plants (Basel). 2024 Jul 14;13(14):1937. doi: 10.3390/plants13141937 (PMC11280814; doi:10.3390/plants13141937)
Supplement: Supplementary file 1 [file plants-13-01937-s001.zip › Table S2.pdf]

Table S2. The results of ANOVA test and Tukey HSD test for the dataset comprising morphological and anatomical features of the floating, submersed leaves, and stems.

	ANOVA		TUKEY HSD test					
**Code**	**F**	**p**	**I**	**II**	**III**	**IV**	**V**	**VI**
Flo1	3.58	0.01	5.51^{b} ± 1.23	4.80^{a} ± 0.73	4.30^{a} ± 0.84	4.55^{ab} ± 0.73	4.59^{ab} ± 0.53	5.03^{ab} ± 0.73
Flo2	1.69	0.15	2.10 ± 0.65	1.94 ± 0.20	1.80 ± 0.30	2.00 ± 0.43	1.99 ± 0.26	2.19 ± 0.22
Flo3	3.30	0.01	8.79^{b} ± 3.98	6.82^{ab} ± 1.65	5.43^{a} ± 1.75	6.67^{ab} ± 2.44	6.54^{ab} ± 1.61	7.76^{ab} ± 1.65
Flo4	3.73	0.00	13.33^{b} ± 2.49	13.16^{ab} ±1.60	10.58^{a} ± 2.59	10.91^{b} ± 3.16	12.12^{ab} ± 1.22	12.64^{ab} ± 0.92
Flo5	3.29	0.01	12.99^{b} ± 3.08	12.98^{a} ± 1.17	11.02^{ab} ± 2.41	9.90^{b} ± 3.56	10.99^{ab} ± 1.43	11.39^{ab} ± 1.37
Flo6	10.81	0.00	336.87^{b} ± 65.17	250.88^{a} ± 34.87	260.37^{a} ± 18.48	250.48^{a}± 60.44	251.47^{a} ± 36.79	208.97^{a}± 39.79
Flo7	7.42	0.00	141.91^{c} ± 30.65	118.90^{ab} ± 19.51	115.62^{bc} ± 27.31	109.18^{bc} ± 30.3	111.06^{b} ± 23.62	80.49^{a} ± 16.80
Flo8	7.18	0.00	204.36^{b} ± 45.46	133.48^{a} ± 27.71	148.27^{a} ± 24.243	140.06^{a} ± 51.47	143.38^{a} ± 27.95	130.52^{a} ± 34.23
Flo9	13.54	0.00	181.95^{c} ± 15.67	168.92^{b} ± 10.87	148.06^{a} ± 10.50	165.24^{bc} ± 15.77	158.26^{ab} ± 16.73	144.00^{a} ± 13.24
Flo10	3.02	0.02	20.69^{b} ± 3.45	19.50^{ab} ± 5.31	21.31^{b} ± 3.72	17.00^{ab} ± 2.79	16.75^{a} ± 4.49	19.08^{ab} ± 3.55
Flo11	13.15	0.00	515.64^{a} ± 29.41	570.38^{a} ± 33.82	588.90^{a} ± 51.57	481.70^{a} ± 75.40	489.49^{a} ± 51.11	478.36^{b} ± 31.69
Flo12	23.18	0.00	32.29^{cd} ± 1.21	33.76^{bc} ± 1.30	34.00^{d} ± 1.73	30.15^{d} ± 1.68	27.81^{ab} ± 4.18	27.42^{a} ± 1.90
Flo13	23.48	0.00	15.98^{ab} ± 0.85	20.11^{a} ± 1.23	20.47^{d} ± 2.06	15.57^{d} ± 1.94	17.86^{c} ± 1.65	17.47^{bc} ± 1.00
Flo14	3.76	0.00	331.07^{b} ± 23.99	319.28^{ab} ± 11.90	318.85^{b} ± 26.44	317.16^{b} ± 28.40	291.16^{a} ± 30.85	317.38^{ab} ± 12.02
Flo15	4.48	0.00	277.68^{ab} ± 23.95	287.55^{a} ± 26.48	291.63^{b} ± 28.17	260.82^{ab} ± 27.65	260.92^{a} ± 26.51	295.21^{b} ± 17.40
Flo16	8.23	0.00	30.75^{c} ± 2.41	28.01^{a} ± 1.59	28.17^{ab} ± 2.24	26.51^{ab} ± 2.39	26.67^{a} ± 1.43	29.43^{bc} ± 1.47
Flo17	6.85	0.00	27.08^{b} ± 1.84	25.46^{a} ± 1.39	25.16^{ab} ± 2.77	23.26^{ab} ± 2.63	23.30^{a} ± 1.35	26.36^{b} ± 1.81
Sub1	4.83	0.00	5.60^{bc} ± 1.87	5.22^{ab} ± 0.88	4.27^{a} ± 0.53	4.49^{abc} ± 0.91	4.54^{ab} ± 0.97	6.05^{c} ± 1.48
Sub2	7.90	0.00	0.59^{ab} ± 0.26	0.70^{ab} ± 0.09	0.52^{a} ± 0.07	0.57^{bc} ± 0.11	0.71^{bc} ± 0.12	0.80^{c} ± 0.11
Sub3	6.70	0.00	2.74^{bc} ±1.67	2.53^{ab} ± 0.65	1.60^{a} ± 0.33	1.82^{abc} ± 0.76	2.53^{abc} ± 0.87	3.52^{c} ± 1.12

Sub4	6.55	0.00	254.54^{bc} ± 32.02	254.52^{ab} ± 40.2	196.37^{a} ± 37.67	218.56^{bc} ± 27.73	264.98^{c} ± 35.76	235.44^{abc} ± 50.93
Sub5	1.85	0.11	47.67 ± 4.74	51.18 ± 17.25	44.85 ± 5.73	54.19 ± 6.23	51.23 ± 8.11	48.80 ± 6.11
Sub6	7.54	0.00	102.16^{c} ± 10.42	98.79^{ab} ± 7.36	79.84^{a} ± 10.44	86.69^{bc} ± 8.92	97.42^{bc} ± 12.67	97.26^{bc} ± 18.22
Sub7	0.16	0.98	265.36 ± 59.07	268.76 ± 45.5	272.24 ± 74.13	280.88 ± 57.35	265.95 ± 64.58	280.12 ± 49.16
Sub8	0.21	0.96	20.48 ± 5.59	19.44 ± 6.46	21.05 ± 6.49	20.70 ± 5.62	19.44 ± 5.35	20.99 ± 5.58
Sub9	1.44	0.22	11.10 ± 3.09	11.36 ± 1.83	11.29 ± 2.76	9.27 ± 2.69	10.87 ± 2.85	9.75 ± 2.20
Ste1	1.39	0.24	6.72 ± 2.62	5.81 ± 1.39	5.13 ± 1.99	5.15 ± 1.66	5.39 ± 1.57	5.11 ± 2.08
Ste2	0.79	0.56	14.49 ± 4.20	13.36 ± 2.34	13.16 ± 3.19	12.43 ± 2.42	13.03 ± 3.11	14.58 ± 4.49
Ste3	3.07	0.01	530.45^{ab} ± 91.08	535.53^{ab} ± 110.13	425.81^{a} ± 58.11	526.32^{ab} ± 85.43	570.79^{b} ± 131.51	595.40^{b} ± 229.90
Ste4	1.36	0.25	9.03 ± 2.95	8.01 ± 3.62	10.28 ± 2.51	10.73 ± 2.43	9.57 ± 1.44	8.93 ± 4.74
Ste5	3.71	0.00	1310.73^{ab} ± 180.72	1428.29^{ab} ± 220.40	1112.49^{a} ± 171.33	1371.21^{b} ± 176.47	1407.89^{b} ± 257.18	1386.26^{b} ± 372.38
Ste6	1.85	0.11	199.41 ± 34.17	231.57 ± 42.49	185.70 ± 49.37	233.46 ± 48.31	188.47 ± 42.28	210.81 ± 97.65

Feature codes according to Table 2.
The results of ANOVA are expressed as F, p values, colored values in red are statistically significant at $p<0.05$
The results of Tukey HSD post hoc test are expressed as means ± standard deviation with statistical differences if $p<0.05$.
Different superscript letters indicate significant differences between means in Tukey test.
