# Supplementary material for: Morphological and Anatomical Differentiation of Potamogeton gramineus in Relation to the Presence of Invasive Species Elodea nuttallii: A Case Study from Vlasina Lake, Serbia
Source: Plants (Basel). 2024 Jul 14;13(14):1937. doi: 10.3390/plants13141937 (PMC11280814; doi:10.3390/plants13141937)
Supplement: Supplementary file 1 [file plants-13-01937-s001.zip › Table S3.pdf]

Table S3. The results of independent samples Studens´s t-test for the dataset comprising all measured morphological and anatomical features of *Potamogeton gramineus*.

Independent samples t-test						
Code	*E. nuttallii*	N	Mean	Std. Dev.	t	p
Flo1	absent	41	4.83	1.05	0.484	0.629
	present	38	4.73	0.69		
Flo2	absent	41	1.94	0.48	-1.508	0.136
	present	38	2.06	0.24		
Flo3	absent	41	6.90	3.07	-0.198	0.844
	present	38	7.00	1.68		
Flo4	absent	41	12.25	2.99	0.715	0.477
	present	38	11.88	1.45		
Flo5	absent	41	12.25	3.10	2.817	**0.006**
	present	38	10.75	1.44		
Flo6	absent	41	280.41	62.42	3.565	**0.001**
	present	38	236.56	41.39		
Flo7	absent	41	124.64	30.87	3.867	**0.000**
	present	38	99.93	24.13		
Flo8	absent	41	160.41	50.16	2.465	**0.016**
	present	38	137.83	29.80		
Flo9	absent	41	165.10	19.75	2.251	**0.027**
	present	38	155.77	16.12		
Flo10	absent	41	20.53	3.37	3.213	**0.002**
	present	38	17.61	4.51		
Flo11	absent	41	560.72	62.60	6.716	**0.000**
	present	38	483.18	38.97		
Flo12	absent	41	33.40	1.71	8.903	**0.000**
	present	38	28.46	2.94		
Flo13	absent	41	19.00	2.63	4.183	**0.000**
	present	38	16.97	1.63		
Flo14	absent	41	322.68	26.36	2.496	**0.015**
	present	38	308.57	23.34		
Flo15	absent	41	286.08	26.80	2.213	**0.030**
	present	38	272.32	28.39		
Flo16	absent	41	28.90	2.60	2.573	**0.012**
	present	38	27.54	1.99		
Flo17	absent	41	25.84	2.56	2.925	**0.005**
	present	38	24.31	2.09		
Sub1	absent	41	4.97	1.28	-0.236	0.814
	present	38	5.04	1.34		
Sub2	absent	41	0.60	0.17	-2.575	**0.012**
	present	38	0.69	0.14		
Sub3	absent	41	2.24	1.11	-1.523	0.132

	present	38	2.63	1.13		
Sub4	absent	41	232.37	43.07	-0.646	0.520
	present	38	238.93	45.92		
Sub5	absent	41	47.77	6.14	-1.770	0.081
	present	38	51.41	11.53		
Sub6	absent	41	92.53	14.06	-0.358	0.721
	present	38	93.69	14.08		
Sub7	absent	41	269.02	63.13	-0.501	0.618
	present	38	275.65	52.63		
Sub8	absent	41	20.35	5.85	-0.018	0.986
	present	38	20.38	5.69		
Sub9	absent	41	11.26	2.77	2.203	**0.031**
	present	38	9.96	2.36		
Ste1	absent	41	5.83	2.16	1.396	0.167
	present	38	5.21	1.66		
Ste2	absent	41	13.63	3.29	0.355	0.724
	present	38	13.35	3.46		
Ste3	absent	41	493.17	92.69	-2.273	**0.027**
	present	38	563.98	164.24		
Ste4	absent	41	9.16	2.73	-0.820	0.415
	present	38	9.75	3.56		
Ste5	absent	41	1275.24	218.50	-1.983	0.051
	present	38	1387.90	283.30		
Ste6	absent	41	204.78	48.10	-0.517	0.606
	present	38	211.55	67.36		

Feature codes according to Table 2.
