# Supplementary material for: Morphological and Anatomical Differentiation of Potamogeton gramineus in Relation to the Presence of Invasive Species Elodea nuttallii: A Case Study from Vlasina Lake, Serbia
Source: Plants (Basel). 2024 Jul 14;13(14):1937. doi: 10.3390/plants13141937 (PMC11280814; doi:10.3390/plants13141937)
Supplement: Supplementary file 1 [file plants-13-01937-s001.zip › Table S4.pdf]

Table S4. The results of Mann Whitney U test comprising the environmental variables measured at sites with and without present *Elodea nuttaliii*.

	E. nuttallii	N	Mean	Std. Dev.	Z	p
Temperature	present	3	21.06	0.64	**-1.964**	**0.050**
	absent	3	23.90	1.93		
pH	present	3	6.54	0.05	**-1.964**	**0.050**
	absent	3	5.99	0.10		
Saturation	present	3	121.13	-1.09	-1.091	0.275
	absent	3	133.10	2.49		
Concentration of O_2	present	3	9.47	1.36	-0.218	0.827
	absent	3	9.63	1.71		
Depth	present	3	61.00	15.39	-1.091	0.275
	absent	3	48.33	15.04		
Conductivity	present	3	85.37	0.51	-1.771	0.077
	absent	3	83.53	1.17		

Bolded values are statistically significant (Z, $p<0.05$).
