# Supplementary figures and images for: Morphological and Anatomical Differentiation of Potamogeton gramineus in Relation to the Presence of Invasive Species Elodea nuttallii: A Case Study from Vlasina Lake, Serbia
Source: Plants (Basel). 2024 Jul 14;13(14):1937. doi: 10.3390/plants13141937 (PMC11280814; doi:10.3390/plants13141937)

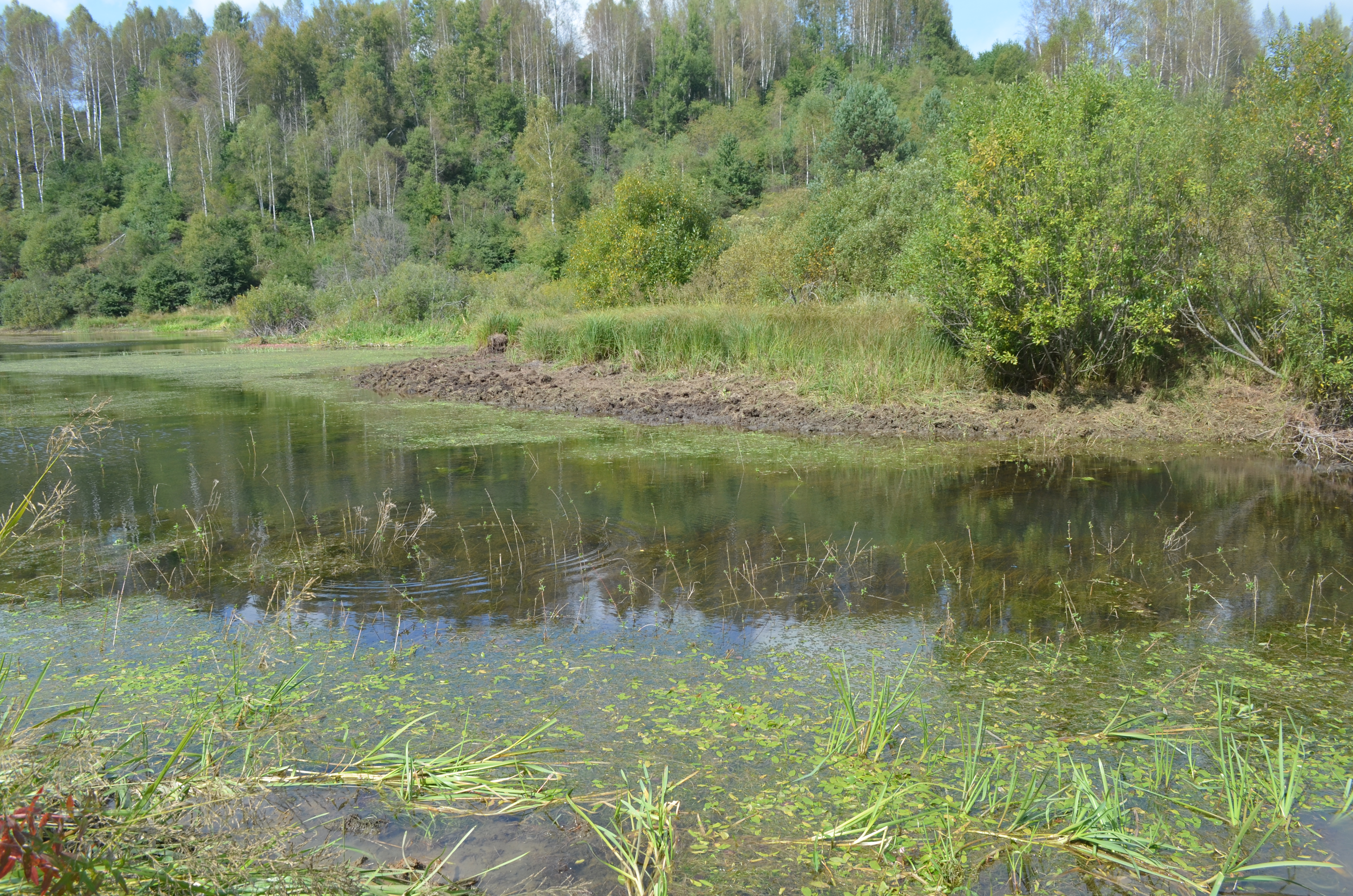

Supplement: Supplementary file 1 [file plants-13-01937-s001.zip › Figure S1.JPG]

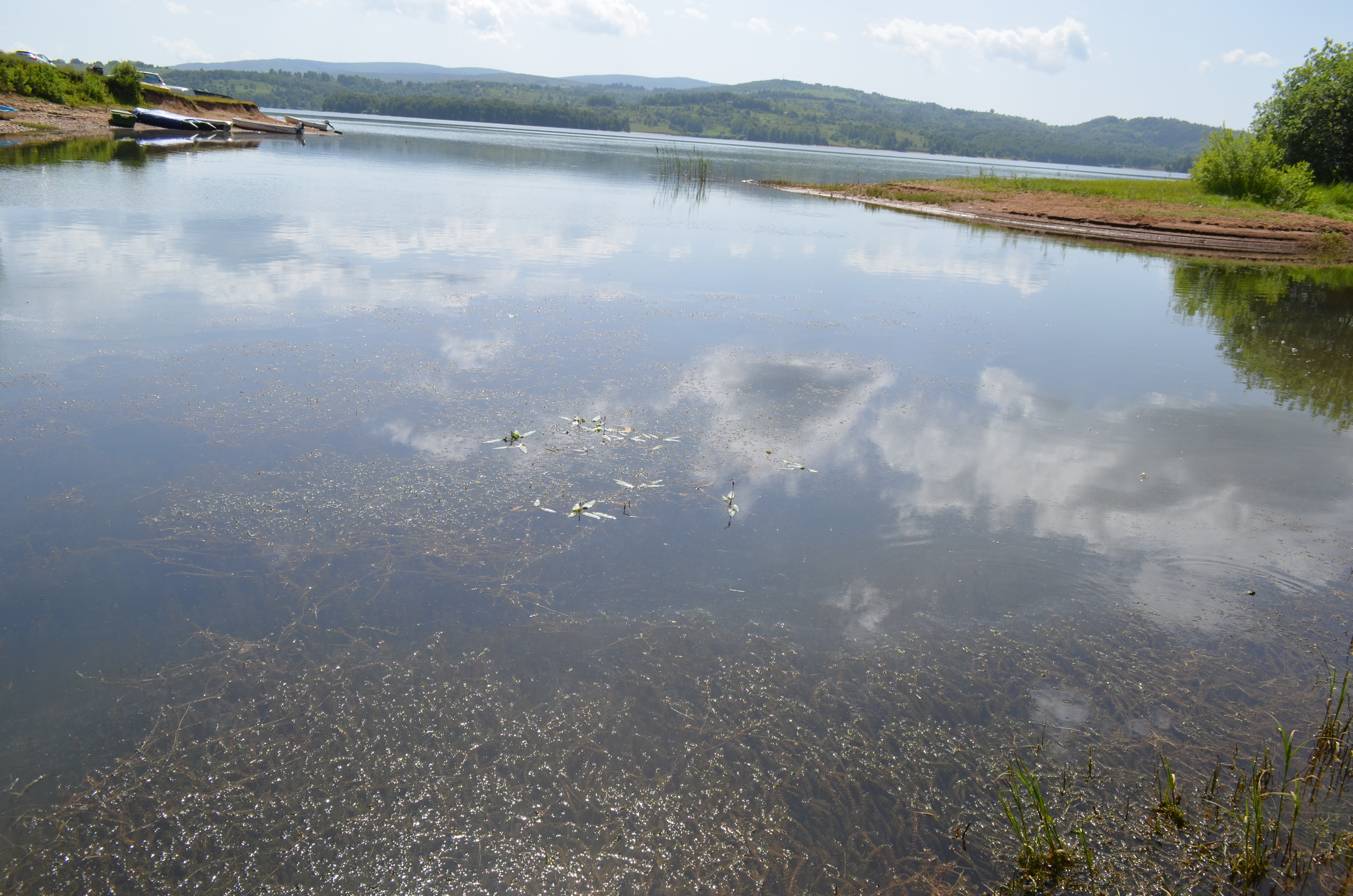

Supplement: Supplementary file 1 [file plants-13-01937-s001.zip › Figure S2.JPG]

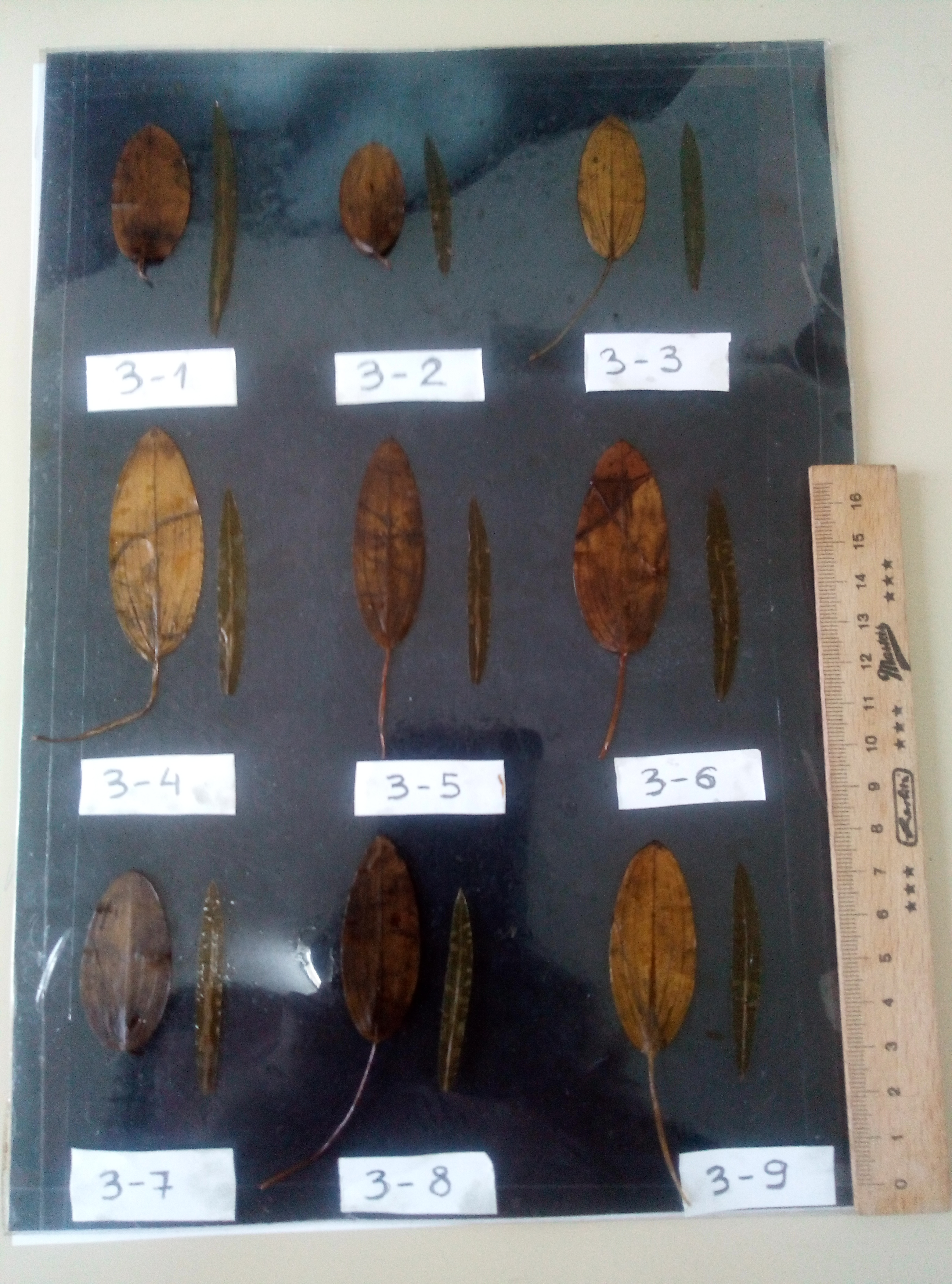

Supplement: Supplementary file 1 [file plants-13-01937-s001.zip › Figure S3.jpg]
